# Detecting and tracking using 2D laser range finders and deep learning

**DOI:** 10.1007/s00521-022-07765-6

**Published:** 2022-09-13

**Authors:** Eugenio Aguirre, Miguel García-Silvente

**Affiliations:** Department of Computer Science and A.I. (DECSAI). Andalusian Research Institute in Data Science and Computational Intelligence (DaSCI). CITIC-UGR., University of Granada (UGR), 18071 Granada, Spain

**Keywords:** People detection and tracking, 2D laser, Deep learning, Machine learning, Automatic labelling

## Abstract

Detecting and tracking people using 2D laser rangefinders (LRFs) is challenging due to the features of the human leg motion, high levels of self-occlusion and the existence of objects which are similar to the human legs. Previous approaches use datasets that are manually labelled with support of images of the scenes. We propose a system with a calibrated monocular camera and 2D LRF mounted on a mobile robot in order to generate a dataset of leg patterns through automatic labelling which is valid to achieve a robust and efficient 2D LRF-based people detector and tracker. First, both images and 2D laser data are recorded during the robot navigation in indoor environments. Second, the people detection boxes and keypoints obtained by a deep learning-based object detector are used to locate both people and their legs on the images. The coordinates frame of 2D laser is extrinsically calibrated to the camera coordinates allowing our system to automatically label the leg instances. The automatically labelled dataset is then used to achieve a leg detector by machine learning techniques. To validate the proposal, the leg detector is used to develop a Kalman filter-based people detection and tracking algorithm which is experimentally assessed. The experimentation shows that the proposed system overcomes the Angus Leigh’s detector and tracker which is considered the state of the art on 2D LRF-based people detector and tracker.

## Introduction

People detection and tracking are key skill for mobile robots in a lot of applications including Human Robot Interaction (HRI) [1], efficient navigation among people [2] or aspects related to the safety when humans and autonomous robots share the same space [3]. In order to carry out these tasks, several approaches and sensory modalities have been used. Mainly, computer vision techniques have been applied depending on the kind of camera: Monocular, Stereo or RGB-Depth. In this area, deep learning techniques allow robust person detection. Reference [4] shows an application of YOLO (You Only Look Once) [5] for people detecting in the aim to identify fallen persons. In other approaches, depth information is also taken into account. For example, in Ref. [6] a social robot uses a Kinect sensor fusing colour and depth information for people detecting. Associated to the RGB-Depth sensors, skeleton-based approaches have been proposed [7, 8] allowing the recognition of person pose and some actions or behaviours. Stereo vision has also been applied to address this task. For example, colour and depth information supplied by stereoscopic vision has been used to propose fuzzy algorithms for detecting and tracking people [9].

Despite the progresses in people detection and tracking by computer vision techniques, 2D LRFs are still present in the sensory system of many mobile robots, especially in the case of social and service robots. The large fields of view, high accuracy and robustness of 2D LRFs make them an interesting sensor for people detection. In contrast, vision-only-based approaches have several drawbacks. Vision-based devices, i.e. monocular, stereo cameras and RGB-Depth sensors, usually have a smaller field of view and light conditions can affect dramatically. In particular, on depth information, it is not always reliable and false positives can arise in skeleton-based methods. In addition, when persons are close to the robot, cameras have important difficulties to detect them.

Other kind of sensors can generate volumetric data, such as 3D Lidar [10], which provides accurate depth measurements of the surrounding environment. However, this sensor is costly and the input resolution is lower than a typical RGB sensor. For all the reasons above shown, 2D LRFs are still present in the most of mobile robots.

In the specialised literature several approaches based on 2D LRFs can be found. Some works are needed to detect movement features [11], but this approach falls whether people do not move. Other approaches divide the range data obtained from sensor in clusters using certain jump distance, compute geometric features and use machine learning techniques to classify the clusters as either person or background [12–14]. These approaches are more robust to the lack of people motions. Other types of works focus the attention on human legs detection computing geometric features of human legs, tracking legs over time and defining persons as pairs of legs [15–17]. The latter approaches usually perform better improving the reliability, especially in cases of self-occlusion. More recent works claim the application of deep learning techniques to generate object detectors in a more direct way from the range data. For example, in Ref. [18] a 2D LRF is used to detect pallets in industrial scenarios through the training of a Faster R-CNN (Region-Based Convolutional Neural Networks). A wheelchair and walker detector based on deep learning is proposed in Ref. [19]. This proposal is improved and extended to people detection in Ref. [20]. The deep learning-based detector is compared to previous detectors achieving good results on the precision-recall curves [20]. However, a whole people detection and tracking system is not developed and tested in real time.

In this work, deep learning is not applied to carry out the people detection from 2D LRF in a straight way but to achieve the automatic labelling of a 2D range dataset recorded during the mobile robot navigation. The automatic labelling of datasets has been applied to a wide variety of domains. An approach to automatically generate labelled network traffic datasets using an unsupervised anomaly-based Intrusion Detection Systems is proposed in Ref. [21]. The approach was empirically proven to be an efficient unsupervised labelling system in that research area. Other application is the identification of web pages relevance. An algorithm to automatically generate high-quality training data-based on the frequency of the document including the entity of interest is proposed in Ref. [22]. Also in Human Activity Recognition, automatic labelling has been applied. In Ref. [23] is proposed an automatic labelling framework to directly annotate unlabelled time series data regarding body-worn sensor-based human activity recognition in laboratory settings.

The rest of this paper is organized as follows. The proposal is briefly described in Sect. 2. Section 3 addresses different deep learning approaches used to object detection and how this field has evolved achieving important successes both on people and keypoints detections. Section 4 describes the hardware and software components of our system. After that, the method proposed in this work for people detection and tracking using laser 2D range data and deep learning-based object detection is explained in Sects. 5 and 6. The experimental work that has been carried out on the different aspects of our proposal is shown in Sect. 7. The conclusions and some ideas on future work are commented in Sect. 8.

## Description of the proposal

The approach proposed in this work has been developed thanks to the previous experience of the authors working on people detection and tracking [24] and 2D range data automatic labelling [25]. On the contrary to these previous works, now a monocular camera is used instead Kinect 1.0 sensor so that depth information from images is not needed. Additionally, the monocular camera does not suffer of the drawbacks of Kinect sensor, which has an infrared camera that can be negatively affected by certain environment lighting conditions. The mobile robot used in our proposal has a LRF sensor which is situated at a height of 30 cm above the floor, allowing the robot to scan the people legs. A Jetson TX2 Developer Kit is located above the LRF and connected to both the sensory system of the robot and LRF. Jetson TX2 features a MIPI CSI camera, which has been intrinsically and extrinsically calibrated to the LRF. Both images and 2D range data, beside their timestamps, odometry data and robot velocities, are stored to on-board SSD drive while the robot navigation is carried out. The robot navigates, both autonomously and manually controlled, through an indoor office-like environment where different persons are walking on. This recorded information can be offline dealt with our proposal in a desktop computer or in a laptop. This fact allows us to apply deep learning algorithms using powerful frameworks as TensorFlow 2 Object Detection API [26]. Several deep learning architectures have been studied for our proposal through a comparative experimentation on people detection in images. In particular, a CenterNet HourGlass104 Keypoints 512x512 detection model [27] pre-trained on the COCO 2017 dataset [28] is used to detect people in the images. Boxes and keypoints are generated by this detection model and this information is added to the dataset. 2D range data are clustered by the jump-distance algorithm and geometrical features of these clusters are computed. The detected keypoints of human legs allow the vision-based leg identification. Through the correspondence between laser data and images, the 2D range data can be automatically labelled. Then a study of several machine learning algorithms is carried out to identify the best machine learning approach to generate a binary classifier for people leg detecting. The leg detector is used to design a Kalman filter-based people detection and tracking algorithm. In order to show the feasibility of our proposal, this algorithm is compared to Leigh’s detector and tracker implemented in ROS [17] which is the current state of the art in people detection and tracking for 2D LRF data. Experimental results show that our people detection and tracking system based on the detector obtained from the automatically labelled dataset overcome the performance of the Leigh’s detector and tracker.

To summarize, the contributions of this work are as follows:

1. To the best of our knowledge, this work is the first application of people detection boxes and keypoints to automatically label a 2D laser range dataset aimed to people detection and tracking through 2D LRF.
2. The automatic labelling of the dataset allows the mobile robot to collect data under real operating conditions so that more robust and efficient detectors can be generated by machine learning techniques.
3. The people detection and tracking algorithm proposed in this work, which uses the detector obtained from the automatically labelled dataset, overcomes the Leigh’s detector and tracker available in ROS.

## Deep learning-based object detection

### Two- and one-stage approaches

People detection in images is usually carried out by object detection methods. The goal of object detection methods is to determine whether there are object instances from a category such as people, animals, bicycles or others, in the image. These methods return the spatial position of the detected objects by addressing their bounding boxes or masks (or both) beside their confidence levels. Object detection has been improved with the help of deep learning techniques, in particular Convolutional Neural Networks (CNN) [29]. These proposals can be roughly classified in two main types, namely two-stage approaches and one-stage approaches.

In the case of two-stage approaches, the object detection task is divided into two stages: extract Regions of Interest (RoIs) and then classify and regress them with the help of a training process on ground truth objects. In particular, Region-Based CNN (R-CNN) [29] uses a selective search method to locate RoIs in the input images. Such RoIs are then classified in an independent way through a Deep Convolutional Neural Network (DCN). The next step in this evolution is the extraction of the RoIs directly from the feature maps, namely Fast-RCNN [30]. In order to improve Fast-RCNN, the concept of Region Proposal Network (RPN) is proposed in Faster-RCNN [31]. The idea is to generate RoIs by regressing the anchor boxes through another network allowing the training end to end. Mask detection represents the next step. The objective is the prediction of both objects and their masks. To achieve this, a mask prediction branch is added on Faster-RCNN generating a new model which is known as Mask-RCNN [32]. Other proposal to improve the two-stage approaches, R-FCN [33], focuses the attention on the use of position-sensitive score maps to replace the fully connected layers. The problem of over-fitting is addressed by Cascade R-CNN [34] through the training of a sequence of detectors while the IoU thresholds are increased.

One-stage approaches remove the RoI extraction process and in a straight way classify and regress the candidate anchor boxes. YOLO is built on darknet neural networks and uses fewer anchor boxes that other approaches to perform regression and classification [35]. The family of YOLO detectors is composed by YOLOv2 [5], YOLOv3 [36] and YOLOv4 [37]. Each model improves the speed-accuracy trade-off of previous models incorporating more anchor boxes, new bounding box regression methods or deeper feature detector networks. On the YOLO detectors family, a new version YOLOv5 is being developed [38]. Other approaches to improve the seed-accuracy trade-off are based on Single Shot Detector (SSD) [39]. The idea is to predict the boundary boxes and the classes directly from feature maps in one single pass. The anchor boxes are densely placed over an input image, and then, features from different convolutional layers are used to regress and classify the anchor boxes. In order to avoid the disadvantages of the use of anchor boxes and bounding box regression, keypoint-based object detection approaches have been proposed, namely CenterNet [27, 40]. These methods detect keypoints such as corners or bounding box centre (or both) to represent each object.

### CenterNet

In the case of CenterNet by Zhou et at. [27], a single point situated at the centre of its bounding box is used to represent an object. The rest of object properties, such as size, pose, dimension and other ones, are regressed from image features. In order to carry out the object detection, a keypoint estimation is applied [41]. More specifically, a heatmap is generated from the input image by using a fully convolutional network. In such heatmap there are peaks which correspond to object centres. The object bounding boxes are predicted for each peak from image features. To train the model, dense supervised learning is used. On the inference process, it is a single network forward-pass, without non-maximal suppression for post-processing.

An important aspect on CenterNet for our proposal is the possibility to estimate multi-person human pose [42]. This estimation is made by the prediction of additional outputs at each centre point. In the case of human pose estimation, the attention is focused on the 2D joint locations which are considered as offsets from the centre. Such 2D joint locations are estimated by using a regression-based one-stage human pose estimator.

CenterNet achieves very good speed-accuracy trade-off on COCO validation for real-time detectors. Thanks to the simplicity of the method, it outperforms the results on speed-accuracy trade-off of FasterRCNN and Yolov3 [27]. When CenterNet is equipped with the keypoint estimation network Hourglass-104 [43] and multi-scale testing, CenterNet achieves 45.1% COCO AP according to the results shown in Ref. [27].

CenterNet has been applied to several research areas. For example, in Ref. [44] a fault diagnosis method on train catenary is developed through analysing of the characteristics of the catenary images. The CenterNet-based approach of Ref. [44] presents higher precision and recall values compared with other detection networks. In Ref. [45], an application on biometric recognition is shown. That cited paper addresses the problems for face recognition when people wear masks due to the coronaviruses pandemic. However, human ear recognition is still possible under these conditions. CenterNet is applied to human ear recognition outperforming other detection methods. In Ref. [46], an automatic detection of vehicle targets based on CenterNet model is proposed. A comparative study with Inception-ResNet-V2 [47] and Efficient-Det [48] is carried out. According to their results, CenterNet improves significantly the average detection accuracy. In Ref. [49], an application on video surveillance is developed. A real-time top view-based person detection system is proposed by using CenterNet for people detection. The model is trained and tested on a top view data set achieving an overall detection accuracy of 95%. A distracted driving detection scheme based on CenterNet is proposed in Ref. [50]. The results demonstrated that the proposed scheme can detect distracted behaviours in real-time while driving with a mean average precision (mAP) of 97.0%, which outperforms some representative detection methods, such as CornerNet, YOLO v3 and YOLO v4.

In our work, a CenterNet model is used to detect people and their keypoints on the images collected in our dataset. Since several CenterNet models exist, an experimental study has been carried out in order to choose the best option for our system. This experimental study is shown in Sect. 7.1. A CenterNet HourGlass104 Keypoints 512 × 512 detection model [27] pre-trained on the COCO 2017 dataset [28] is chosen according to our experimental results.

## System overview

**Fig. 1:**
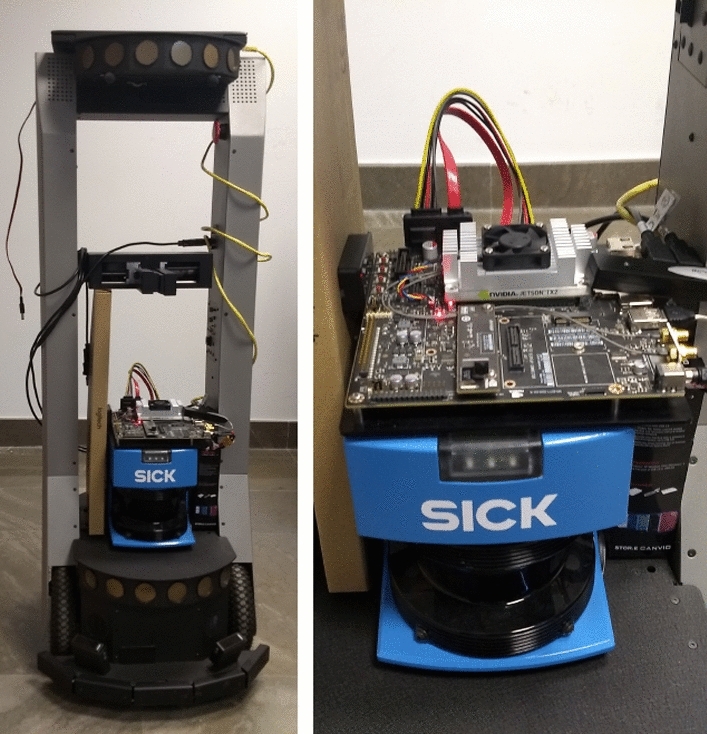
PeopleBot robot equipped with LRF SICK LMS200 and a Jetson TX2 Developer Kit

The hardware of our system has three components. First, the mobile robot which is a PeopleBot mobile robot [51] equipped with a LRF SICK LMS200 [52]. The LRF has a 180$^{\circ }$ field of view, and it can be configured to work at different distances. In the human–robot interaction tasks, the ranges of distances are not usually very high due to the persons stay in the robot surroundings. Thus, in this work the maximum range of distance for the laser measures is 8 metres. Considering this range, the systematic error on the measures is $\pm 15$ mm which is low. Therefore, the 2D range data allow to the robot to achieve measures sufficiently accurate. The second hardware component is an NVIDIA Jetson TX2 Developer Kit which is situated on top of the LRF. This embedded computing board houses both the graphics processing unit (GPU) and central processing unit (CPU) on the same chip. It achieves both an high power efficiency and high processing power. Jetson TX2 incorporates an on-board camera that allows different resolutions depending on the frame rate. In this work, the on-board camera is configured to 640 x 480 resolution at 30 frames per second. Jetson TX2 runs Ubuntu 18.04, and it is connected to both the LRF sensor and the sensory system of PeopleBot robot through USB ports. The third hardware component is a laptop which is situated on top of the robot, and it is connected via ethernet to the Jetson TX2. The function of the laptop is only to connect through ssh protocol to the Jetson TX2 being the interface between the user and the Jetson TX2. Figure 1 shows the hardware components of our system.

**Fig. 2:**
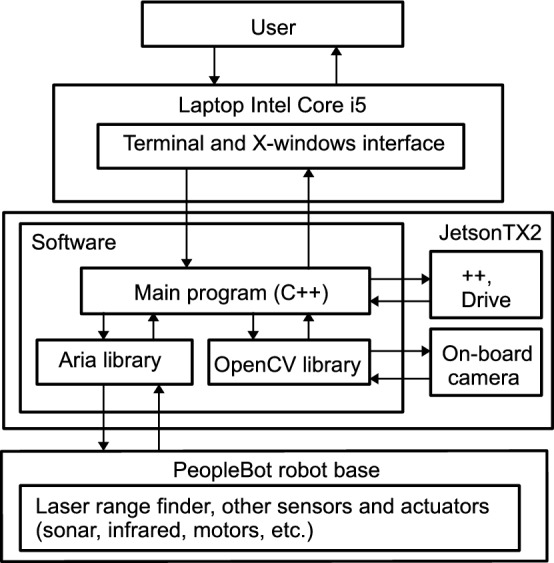
Architecture of proposed system

Regarding the software architecture of our system, it has been implemented using C++ programming language and the resources of the libraries of the robot manufacturer on linux (Ubuntu distribution). The manufacturer supplies this robot with the Aria and ArNetworking libraries. The former is used to execute programs within an on-board computer, while the latter is used to execute client–server programs. The connection between Jetson TX2 and the robot is straight and wired through the USB ports of Jetson TX2. Therefore, Aria library can be used and ArNetworking is not needed. In this way, Jetson TX2 replaces the robot on-board computer which is obsolete and has low computational power. Jetson TX2 is powered by a LiPo battery similar to the batteries used by drones, while the robot is powered by its own plumb battery system. Thanks to the wired connection to LRF, Jetson TX2 can obtain the laser measures in a straight way. The images of Jetson TX2 on-board camera are managed through OpenCV library and both laser measures and corresponding images can be saved to an SSD drive connected to Jetson TX2. Figure 2 shows the architecture of our system.

The people detection and tracking problem can be separated into two subproblems: first, detection; second, tracking. Sections 5 and 6, respectively, explain how such subproblems are solved in our proposal.

## Leg detection using 2D LRF and object-keypoints detectors

### Automatic labelling using bonding box and keypoints

The idea of generating human leg detectors from 2D range data is not new. Several approaches can be found in the specialised literature, as it was addressed in Sect. 1. Machine learning techniques are usually applied to a 2D range dataset which contains instances of legs and background detections. The quality and generalization skills of the detectors trained from these datasets depend on the ability of the instances to represent, in a right way, the sensing of the robot under real-world operating conditions. More specifically, a people detector trained with a poor dataset, which containing an important number of wrong labels or with low level of representativeness, can achieve good values of precision and recall on certain test sets but likely, such detector does not work fine in the real world. In our proposal, a pre-trained CenterNet HourGlass104 Keypoints (CHK) detection model is used to identify people bounding boxes and keypoints to achieve the automatic labelling of the 2D range data as it is explained following.

Our framework relies on 2D range data, existing pre-trained object detectors and a mapping between 2D laser coordinates frame and corresponding images. Now the necessary concepts and notations are introduced.

**Fig. 3:**
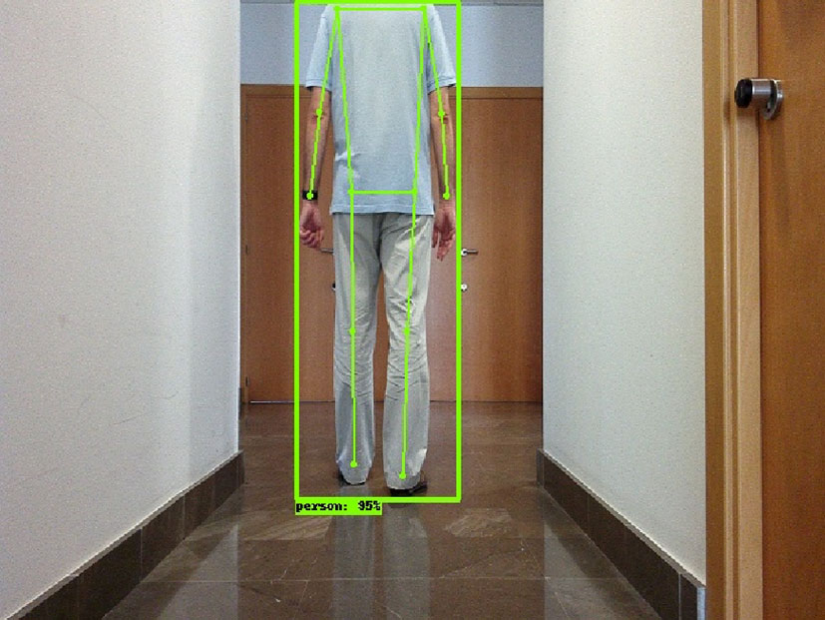
Bounding box and keypoints supplied by CenterNet HourGlass104 Keypoints detector

Let *rc* be the 3D robot coordinates frame. Let *IM* be an on-board camera image. The location of camera is known on the 3D robot coordinates frame *rc*. An object detector aims to localize and classify all objects in *IM*. In this work, the attention is focused only on the objects of class “person”. Let $b_i \in R^4$ for $i=1 \dots P$ be a bounding box that describes a person location in *IM* and being *P* the number of persons detected in *IM*. A person class score $s(b_i)$ predicts the likelihood of detection $b_i$ to be of class “person”. An set of keypoints $KP_i=\{kp_j^i\}$ for $j=1 \dots 16$ is estimated by the detector for every $b_i$. CHK can estimate until 16 keypoints of the human body. In order to identify the human legs only the keypoints corresponding to legs are needed. Such keypoints are $kp_{13}^i$ and $kp_{15}^i$ for left leg of $b_i$ and $kp_{14}^i$ and $kp_{16}^i$ for the right leg of $b_i$. Figure 3 shows an example of bounding box $b_i$ and keypoints $KP_i$ with their segments.

Let $p=\{x,y\}$ be a 2D point sensed by the LRF expressed in laser coordinates. A laser scan $S=\{p_l\}$ for $l=1 \dots L$, being *L* the number of scanned points, can be clustered by using the well-known algorithm of jump distance to generate a set of clusters $C =\{c_m\}$ for $m=1 \dots M$, being *M* the number of clusters, where each $c_m=\{p_n\} \mid p_n \in S$ for $n=1 \dots N$ being *N* the number of points of $c_m$. The set of clusters *C* is computed taking into account a minimum and maximum value of *N* in order to $c_m$ can be considered a valid cluster. The projection from the 2D laser coordinates into an image coordinates involves a transformation from the LRF measurement to the camera frame and a perspective projection from the camera frame into image coordinates. Let $T_{rc \leftarrow lc}$ be the transformation from the LRF sensor coordinates to the reference frame of the robot. Let $T_{cc \leftarrow rc}$ be the transformation from the robot reference frame to the camera coordinates. The transformation from the LRF to camera coordinates is then defined by

1 $$\begin{aligned} T_{cc \leftarrow lc}=T_{cc \leftarrow rc}T_{rc \leftarrow lc}. \end{aligned}$$

**Fig. 4:**
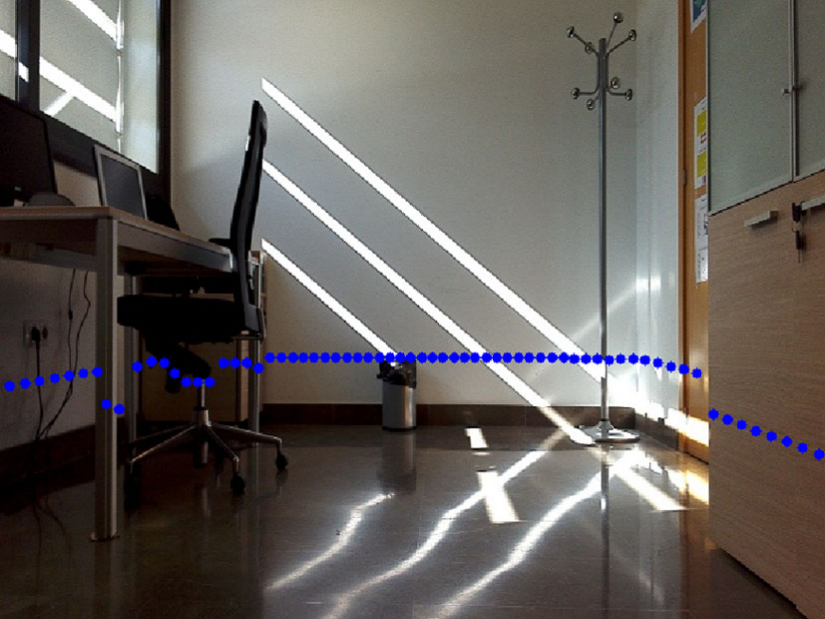
Projection of 2D laser range data to colour image

Finally, by using the projection matrix of the on-board camera defined by the camera intrinsic, $T_{cc \leftarrow lc}$ is projected on the colour image achieving a set of pixels $PI=\{(px_q,py_q)\}$ for $q=1 \dots Q$ being *Q* the number of pixels of the projection. The intrinsic parameters of the on-board camera have been achieved by using the Robust Automatic Detection of Calibration Chessboards approach[53]. Results of the projection of a laser scan *S* to the colour image *IM* can be seen in Fig. 4. The blue points are the laser measures translated to the set of pixels *PI*.

**Fig. 5:**
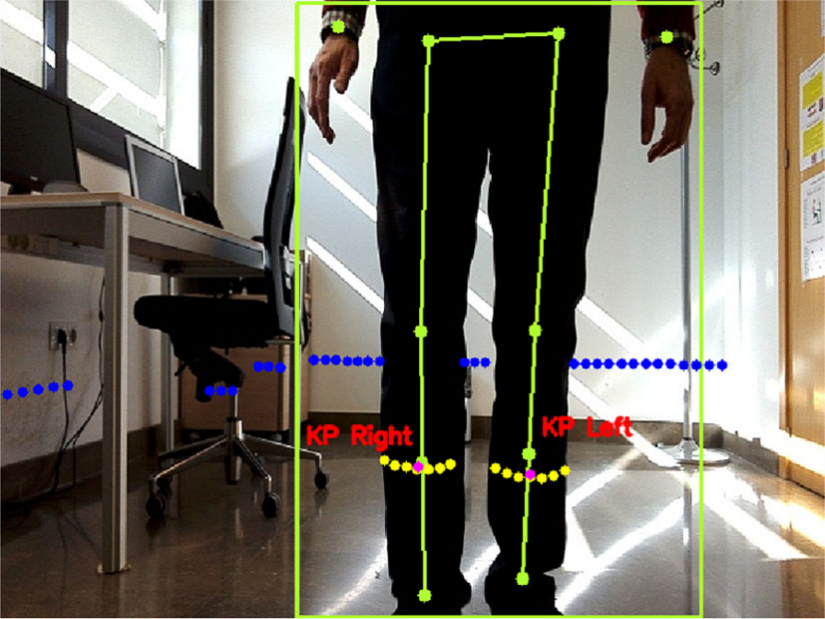
Assignment of the projections of clusters to detect legs

Let $\alpha \in [0.1]$ be a threshold of confidence for the person class. For every $b_i \in IM \mid s(b_i)>\alpha$ two new keypoints $kp_{left}^i$ and $kp_{right}^i$, corresponding to the left and right human legs located within $b_i$, are computed from $KP_i$. $kp_{left}^i$ is the middle point of $kp_{13}^i$ and $kp_{15}^i$, and $kp_{right}^i$ is the middle point of $kp_{14}^i$ and $kp_{16}^i$. Figure 5 shows examples of $kp_{left}^i$ and $kp_{right}^i$. Following, the process to assign a cluster of *C* to some $kp_{left}^i$ or $kp_{right}^i$ of *IM* is explained for a certain $kp_{left}^i$ since it is similar for the rest.

Given a $kp_{left}^i$ in the image, certain cluster $c_x \in C$ can be assigned to $kp_{left}^i$ in the following way. For each $c_m \in C$ the centroid $t_m$ of $c_m$ is computed. Each centroid $t_m$ is a 2D point in the laser coordinates therefore it must be mapped to the image *IM*. Let $t'_m$ and $c'_m$ be the mapping of $t_m$ and $c_m$ in the image respectively, the distance in pixels from $t'_m$ to $kp_{left}^i$ can be computed. Let $c_x$ be the cluster such that its centroid mapping $t'_x$ is the nearest to $kp_{left}^i$, the distance $d_{min}$ from $t'_x$ to $kp_{left}^i$ must be under certain threshold $\beta$. $\beta$ is measured in pixels and it is computed considering the height of the impact of the laser in the human legs and the distance of the robot to $t_m$. The closer $t_m$ is, the bigger $\beta$ must be. On the contrary, the faraway $t_m$ is, the lower $\beta$ must be. If $d_{min} < \beta$, the rest of points of $c'_x$ have to be checked to assure whether they are also under certain distance threshold $\gamma$ measured in pixels. This checking operation is similar to the case of the centroid, but it is needed to assure that the cluster $c_x$ is definitively assigned to $kp_{left}^i$ and therefore it can be labelled as a real human leg. Once $c_x$ is assigned to a particular $kp_{left}^i$, such $kp_{left}^i$ cannot be used again in the assignment process. Figure 5 shows the results of this assignment process. Yellow points are the projections in the image of the 2D points of the clusters assigned to $kp_{left}^i$ and $kp_{right}^i$. Magenta points are the projections of corresponding centroids $t_m$. Blue points are the projections of the rest of clusters.

The assignment process explained above allows the automatic labelling of clusters for class “leg” from our raw 2D range dataset. Additionally in the cases of both human legs are assigned, the centroids of such legs are used to set ground truth positions considering the middle point of centroids in laser coordinates as the ground truth of a person. Then, clusters labelled as leg are stored in a clusters dataset and they are considered like positive instances to train a binary classifier. The negative instances are clusters belonging to background or to objects of classes which are different to person. Negatives instances are properly added to the clusters dataset to achieve both training and validation sets with a balanced number of instances. The use of machine learning to achieve the classifier is explained in the following.

### Features extraction to achieve a set of examples

**Fig. 6:**
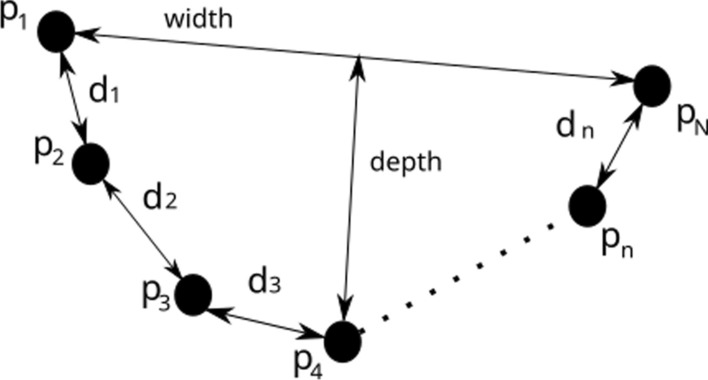
Geometrical features used to represent a cluster

Given the clusters dataset achieved in Sect. 5.1, it is needed the representation of each cluster by a set of representative geometrical features. There are different proposals in previous works that have achieved good results. The Leigh’s detector implemented in ROS [17] uses 15 features such as number of points, linearity, circularity, width, length and boundary length, among others. In our proposal, we use the features proposed by [54], namely the *contour* of the neighbouring points, the *width* and the *depth*, which basically coincide with the three latter features of Leigh’s detector. Let $c_m=\{p_n\} \mid p_n \in S$ for $n=1 \dots N$ be a cluster, the *contour* is computed summing the distances among its points, taking the points by couples, specifically $p_1$ to $p_2$, $p_2$ to $p_3$ and so on until $p_{N-1}$ to $p_{N}$. The distance from point $p_1$ to point $p_N$ is the *width*, while the distance from the furthest point to the line formed by $\overline{p_1p_N}$ is the *depth*. These geometrical features are shown in Fig. 6.

By using this process of feature extraction besides label of each class, leg or non-leg, the clusters dataset is transformed to a set of positive and negative examples which can be used by typical machine learning algorithms. The set of examples counts with 2316 positive instances, examples labelled as leg, and the same number of negative instances, examples labelled as non-leg. In total they sum 4632 examples achieving a balanced set of examples. In order to choose the more interesting machine learning algorithm for this kind of dataset, an experimental study has been carried out which is explained in Sect. 7.2. The classifier achieved by using the algorithm addressed in Sect. 7.2 is used in our proposal to solve the leg detection from 2D range data.

## People tracking

Once leg detection from 2D LRF data has been solved by our classifier trained from the automatically labelled clusters dataset, the second component of our system is now explained. People tracking is achieved using both the Kalman filter [55] and the Global Nearest Neighbour (GNN) method. Former is used to predict and deal with the uncertainty of the people motion over consecutive laser scans and the latter to resolve the data association problem between such consecutive scans.

To track the people, first some initiation conditions are needed. The detected legs in a scan must be grouped in pairs of legs depending on the distance among them. Each pair of associated legs is a candidate to person which position is the middle-point of the centroids of the legs. The candidates to person have to be detected in a minimum number of consecutive scans to become a detected person. Then, a tracker is associated with the detected person. Once a tracker has been assigned to a person, it is not needed the detection of both legs in every scan. In particular, there are situations in which only one leg can be detected. Such situations are generated due to occlusions usually produced by the person motion. In these cases, the observed position will be the centroid of the unique detected leg. Kalman filter is able to deal with these situations properly to generate the estimated person position in each scan even under occlusion situations and temporal lack of leg sensing during certain time. In the following, the Kalman filter-based people tracking algorithm of our proposal is described.

Detected people are considered as observations for the Kalman filter. The observations at time *k* are denoted as $z_k=\{z_k^1, z_k^2,\dots , z_k^{M_k}\}$ where $M_k$ is the total number of detected people at time *k*. The position of all detected people is individually tracked over time using a Kalman filter for every person. Let $x_k^j$ be a track of a person *j* at time *k*, the set of all active tracks is denoted as $X_k=\{x_k^1, x_k^2, \dots , x_k^{N_k}\}$ where $N_k$ is the total numbers of tracks at time *k*. The state vector for each track $x_k^j=[x \, y \, \dot{x} \, \dot{y}]^T$ contains the position and velocity of the tracked person in 2D lase coordinates. When a track is initialized, its velocity is zero, while for existing tracks their velocities are properly updated. Regarding the process noise *w* is assumed to be Gaussian with diagonal covariance $Q=qI$ where *I* is the identity matrix.

The observation matrix $H_k$ includes the position of the person at time *k*. The observation noise $v_k$ is also assumed to be Gaussian with diagonal covariance $R_k=rI$. Due the laser measures are very accurate, *r* can be setup to a low value. Since several persons can be tracked over the time, it is necessary to set a correspondence among the detections $z_k$ and the previous tracks $X_{k-1}$ in order to generate the updated tracks $X_k$ for the current time *k*. This is addressed by using a GNN data association method which is solved via the Munkres assignment algorithm [56].

People tracking is an iterative process which includes several steps. First, the state estimates $X_{k \mid k-1}$ for time *k* are evolved from the tracks $X_{k-1}$ at time $k-1$ according to the well-known Kalman filter equations. Then, the Mahalanobis distance between the detection $z_k^i$ and the propagated track $x_{k \mid k-1}^j$ is used to compute the Munkres cost matrix. The covariance applied to calculate the Mahalanobis distance is the innovation covariance $S_k^j$ achieved by the linear Kalman filter equation, $S_k^j=H_k P_{k \mid k-1}^j H_k^T + R_k$ where $P_{k \mid k-1}^j$ is the prediction covariance of the propagated track $x_{k \mid k-1}^j$. Once the assignment is accomplished, the information on each detected people is used to achieve the updated track position $x_k^j$ via the Kalman filter equations. Two typical situations have to be considered in this iterative process. On the one hand, tracks without matched detections can exist. Such tracks have to be propagated forward without observation updates and their innovation covariances *S* increase until certain threshold. If that threshold is achieved, then the person track is deleted. On the other hand, detections without matched tracks can arise. In these cases, such tracks are candidate to become new people tracked whether the initiation conditions are accomplished.

Following, Sect. 7 shows the experimental work that has been carried out on different aspects of our proposal.

## Experimental results

### Comparative study on CenterNet architectures

In the TensorFlow 2 Detection Model Zoo, several CenterNet models pre-trained on the COCO 2017 dataset can be found. These models have different speed, COCO mAP and outputs. Only four models allow both boxes and keypoints detections. These models are equipped with HourGlass104, Resnet50 V1 FPN or Resnet50 V2 architectures. The sizes of input images can be 1024 x 1024 or 512 x 512. In order to choose the best option for our system, a comparative experimental study is carried out. In our system, both people and keypoints detections are needed so that only the four models which generate both boxes and keypoint have been taken into account. Table 1 shows the four CenterNet models analysed.

**Table 1:** CenterNet models pre-trained on COCO 2017 dataset, including an identification code and network architecture

Code	Model + architecture + input image size
1	CenterNet HourGlass104 512 × 512
2	CenterNet HourGlass104 1024 × 1024
3	CenterNet Resnet50 V1 FPN 512 × 512
4	CenterNet Resnet50 V2 512 × 512

The evaluation measures for this experimental study are computed using only the images containing instances of person class in the COCO 2017 validation set. This experimental study performs COCO-style evaluation on the given images so that predicted objects are matched to ground truth objects in descending order of confidence with matches requiring a minimum IoU of 0.5. Thus, the computed measures are mAP, Precision, Recall and F1-score for IoU=0.5. Computation time for image in milliseconds is also shown. The experimentation has been run in a laptop with Ubuntu 20.04 using Tensorflow 2 on GPU Nvidia GeForce RTX 2070 Super 8 GB memory. Table 2 shows the people detection results of the models listed in Table 1 on the person class of COCO 2017 validation set (2693 images containing at least one person).The best speed-accuracy trade-off is printed in bold in the Table 2.

**Table 2:** Results of CenterNet models and architectures indicated in Table 1

Code	mAP	Prec.	Recall	F1	Time ms
**1**	**0.32**	**0.82**	**0.81**	**0.81**	**384**
2	0.33	0.83	0.81	0.82	625
3	0.23	0.80	0.75	0.77	312
4	0.22	0.80	0.68	0.73	312

**Fig. 7:**
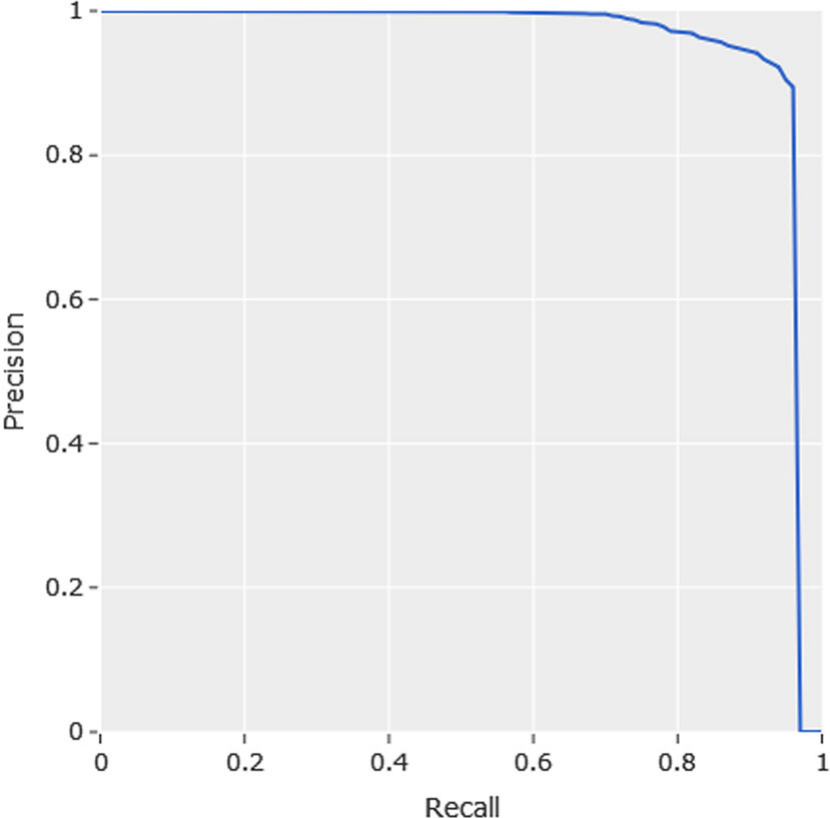
Precision–recall curve for CenterNet HourGlass104 512 × 512 on our images dataset

The best speed-accuracy trade-off is achieved by model Code 1, namely CenterNet HourGlass104 512 × 512. As it was indicated above, the results of Table 2 have been computed on the person class of the COCO 2017 validation set. In such validation set, the images containing people are not focused on a particular application. However, in our work, the people detection is carried out by the Jetson TX2 on-board camera which is located at approximately 30 centimetres on the floor. In our system, the camera captures the people images taking the legs and the rest of the people body whether the person is located to a certain distance of the robot. In order to take into consideration such particularities of our images, the performance of CenterNet HourGlass104 512 × 512 has been tested on our people images dataset. This dataset contains 1607 images which have been taken from the point of view of our robot in office-like indoor environments. The results of people detection using CenterNet HourGlass104 512 × 512 on our images dataset are: $mAP=0.79$, $Precision=0.87$, $Recall=0.97$, $F1=0.92$. These results have been computed using $IoU=0.5$ and following the COCO-style evaluation. Precision-recall curve can be computed depending on confidence threshold to consider a prediction with at least $IoU=0.5$ as true positive. Figure 7 shows the precision–recall curve of people predictions on our images dataset.

By examining the precision–recall curve of Fig. 7, the best precision–recall trade-off is achieved for $Precision=0.927$ and $Recall=0.93$ which correspond to $Confidence=0.362$. Thus, using this level of confidence as threshold, an high number of true positives (TP) and low level both of false positives (FP) and false negatives (FN) are achieved. Concluding, this detection model is adequate taking into account the particularities of our system and for this reason we have considered it in our proposal. In order to identify the corresponding people keypoints, the version including keypoints detection is used. More specifically, the pre-trained model chosen in this work is CenterNet HourGlass104 Keypoints 512 × 512 which can be found at TensorFlow 2 Detection Model Zoo.

### Comparative study on machine learning algorithms

Given the balanced set of examples of leg and non-leg achieved in Sect. 5.1, this set is divided in training and test sets. Approximately the 80% of examples are considered for training and 20% for testing. Thus, for each class, there are 1850 instances for training and 466 for testing. To identify the most interesting algorithm for our set of examples, the machine learning platform Weka [57] is used. The checked algorithms are: a Bayesian naive classifier, a Multilayer Perceptron (neural networks), a Support Vector Machine (SVM)-based classifier, a rule-based algorithm (PART), a decision tree C4.5-based algorithm (J48) and a random trees forest-based algorithm (Random Forest). Table 3 shows the average accuracy on 10 runs taking into account tenfold cross-validation in each run. The results are ascendingly ordered by accuracy average. The accuracy is considered as the percentage of correctly classified instances (both positives and negatives). The best result is printed in bold in the Table 3.

**Table 3:** Accuracy average and standard deviation of several machine learning algorithms

Algorithm	Accuracy average	Std. deviation
Bayes Naive	82.01	1.77
Multilayer perceptron	88.94	1.69
libSVM	90.15	1.49
PART	91.05	1.89
J48	92.58	1.31
** Random forest**	**96.63**	**0.98**

The algorithm with the best result in this experiment is Random Forest so that this algorithm is chosen to build our final classifier. Such classifier is built by a new training process using the previous training set, and it is assessed not only in the training set by tenfold cross-validation but also in the test set. The test set contains examples which have not been used in the training process so that the prediction skills of our classifier are properly tested. Furthermore *Precision*, *Recall* and $F_1$ score have been computed to check the influence of both false positives and false negatives in the behaviour of our classifier. Table 4 shows the results of our final classifier on the training and test sets, respectively.

**Table 4:** Results of leg detection training and test sets

Set	Accuracy	Precision	Recall	$F_1$
Training	0.964	0.964	0.964	0.964
Test	0.905	0.912	0.905	0.904

The results are sufficiently good on both the training and test sets so that a binary classifier has been achieved able to detect human legs from 2D LRF data.

### Experimental validation on people detection and tracking

In order to validate our proposal, which we call Keypoints-based People Detector and Tracker (KPDT), a comparative study is carried out using as baseline the detector and tracker by Angus Leigh et al. [17], which we call ROS-based People Detector and Tracker (RPDT). A dataset composed by 2D laser range data and images besides other data as robot position and velocities is built. This dataset is collected from the robot navigating in office-like indoor environments. The robot moves, both autonomously and manually controlled, by different kinds of environments and travelling on both cluttered labs and hallways. In these environments, several people are present to test the skill of the robot to detect them and track their motions on the laser coordinates. To simulate person-following situations, the robot is manually controlled, while the person to be followed was asked to walk naturally and stop periodically to interact with other persons or objects in the environment.

The collected dataset is divided in two parts. First part is used in our proposal to achieve the automatically labelled dataset and the people detection and tracking method as it has been shown in Sects. 5 and 6. To compare our method to RPDT, it is needed to retrain RPDT using the same dataset of positive and negative examples used to train our detector. This training is possible since RPDT is publicly available at [58] including the instructions to retraining the leg detector. The second part of the collected dataset is used to purpose of the comparative evaluation described in this subsection.

The metrics for evaluating both people detection and tracking methods are the commonly used metrics for multi-object tracking, i.e. CLEAR MOT metrics [59]. The tracking performance can be expressed in two numbers: the “tracking precision” which expresses how well the exact positions of persons are estimated, and the “tracking accuracy” which shows how many mistakes the tracker made in terms of misses, false positives and changes of tracked targets (mismatches). Misses arise when a ground truth of a person exists but no estimated person is matched to it. Misses concept is similar to the false negative concept. False positives arise when there are estimated person positions for which no real person exists. Since each person track is identified by an unique identifier ID, mismatches arise when the ID changes over the different frames. This problem is given when two persons are swapped as they pass close to each other. Also, a change of ID is possible when the person is lost because of occlusion and such ID is reset.

Tracking precision is measured by the Multiple Object Tracking Precision (MOTP), which is defined by Equation 2 as

2 $$\begin{aligned} {\rm MOTP}=\frac{\sum _{i,k}d_k^i}{\sum _kc_k} \end{aligned}$$

where $d_k^i$ and $c_k$ are the error in the estimated position of the *i*th person and the number of matching made between estimated people positions and ground truth positions, respectively, at time *k*. Therefore *MOTP* addresses the total error in estimated positions for matched ground truth positions over all frames, averaged by the total number of matches made. It shows the ability of the tracker to estimate precise people positions.

Tracking accuracy is measured by the Multiple Object Tracking Accuracy (MOTA), which is defined by Equation 3 as

3 $$\begin{aligned} {\rm MOTA}=1 - \frac{\sum _k( mi_k + FP_k + sw_k )}{\sum _kg_k} \end{aligned}$$

where $mi_k$, $FP_k$, $sw_k$ and $g_k$ are the number of misses, false positives, mismatches and ground truth values, respectively, at time *k*.

**Fig. 8:**
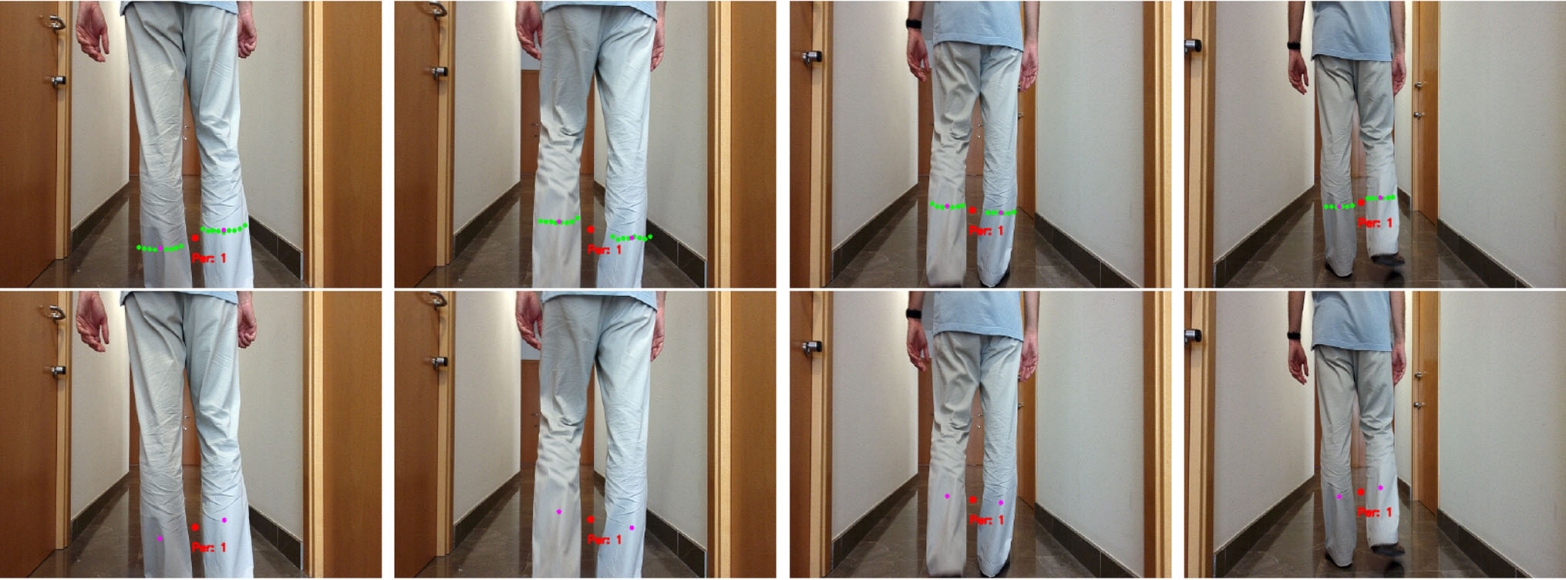
Results in a hallway environment. Upper row shows the KPDT results while bottom row shows the RPDT results

A qualitative comparison on two kinds of environments is first shown. Figure 8 shows the results of both methods while a person is followed by the robot in a hallway. Upper row shows the results of KPDT, and bottom row shows the results of RPDT. The images are commented from left to right in Fig. 8, and they form part of a higher sequence. Our detector KPDT gives as outputs the clusters classified as leg (green points) and their centroids (magenta points). Person position is properly tracked over the frames maintaining the track of the person represented by a red colour point with ID 1. The detector RPDT gives only the centroids (magenta points) of detected legs and the track of the person position labelled as well as ID 1 in a red colour point. In this environment, the results of both methods are very similar.

**Fig. 9:**
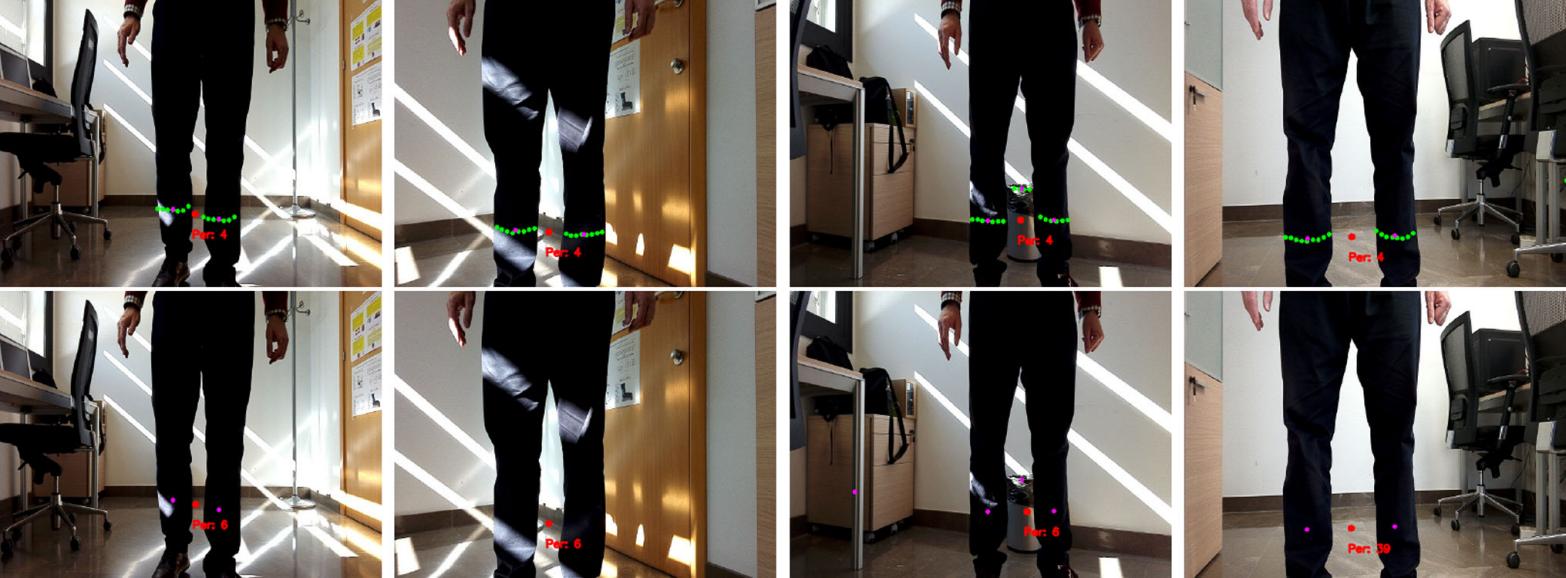
Results in a cluttered environment. Upper row shows the KPDT results, while bottom row shows the RPDT results

Figure 9 shows the results of both methods while a person is followed by the robot in a cluttered environment, in particular, an office. Again, the images are commented from left to right in Fig. 9 and they form part of a higher sequence. First frame shows a person detection with ID 4 by KPDT and ID 6 by the RPDT. Second frame shows a situation where KPDT has detected both legs but RPDT fails. However, the position of person is maintained due to the tracking process. Third frame addresses a situation where the brush is detected as a leg by both detectors although the person position is well maintained in both cases. RPDT achieves one false positive on leg detection more than KPDT since the leg of the table is confused with an human leg. The last frame shows the result after the person moved faster. KPDT was able to maintain the ID for the person, but RPDT needed a reset and a new ID was assigned to the tracked person. In this kind of environment, KPDT shows a performance better than RPDT.

In order to carry out a quantitative comparison, an extensive experimentation is made. The evaluation measures MOTA and MOTP are computed from the results of both methods on different environments including hallways and cluttered environments. Since MOTA is the result of the aggregation of three types of errors, it is interesting to show the raw count of each error type alongside the MOTA score. Table 5 shows the values achieved taking into consideration 3158 ground truth values.

**Table 5:** Results of evaluation measures on both people detection and tracking methods

Method	TP	Miss	FP	Mism	MOTA	MOTP (mm)
KPDT	3068	90	6	0	0.97	19
RPDT	3013	145	36	4	0.94	41

In Table 5, TP column shows the true positive on people tracked and Miss, FP, Mism columns show the number of misses, false positives and mismatches, respectively. Finally, MOTA and MOTP (mm) values are shown. Our proposal has generated a people detector and tracker KPDT which overcomes the results of people detector and tracker by Angus Leigh, RPDT. Our detector KPDT achieves a value of true positive higher while misses, false positives and mismatches are lower. Regarding the MOTP measure, both trackers are precise enough, but the best result is again achieved by KPDT. The average runtime over the dataset of each method has been computed using a laptop with an Intel Core i5 CPU. Only the parts of code related to people detection and tracking haven been taken into account to measure both runtimes. KPDT achieves an average runtime of 3,4 ms, while RPDT achieves 10 ms. Both runtimes allow to process the laser and image data at 25Hz and therefore are suitable to be used at real time being KPDT faster than RPDT.

## Conclusions

In this work, a new method for people detection and tracking using 2D laser range data and deep learning-based object detection has been proposed. On the contrary to previous approaches which uses 2D laser range data manually labelled, in our proposal the range data are automatically labelled with the support of people detection bounding boxes and keypoints. 2D laser data are clustered and the clusters which correspond to human legs are identified through the assignment of the projections of clusters to legs’ keypoints. The automatically labelled clusters dataset is transformed on a set of positive and negative examples by a features extraction process based on geometrical features. The set of examples is used to carry out a comparative study on machine learning algorithms to achieve a robust and efficient human leg detector. This leg detector is used as observation model for a Kalman filter-based people detector and tracker. The feasibility of our proposal has been validated through a comparative study which uses as baseline the people detector and tracker by Angus Leigh et al. available in ROS. Qualitative and quantitative results of such experimental study show that our people detector and tracker overcomes the baseline method achieving a robust and efficient people detection and tracking under real-world operating conditions.

As future works this approach can be used to detect through 2D laser range data other classes of objects which are present in the office-like environments. Also, the people detector and tracker can be useful to analyse the walking of elderly people in order to detect possible problems and supply them help from a service mobile robot.

## Data Availability

The datasets generated during and/or analysed during the current study are available from the corresponding author on reasonable request.

## References

[1] Martinez-Martin E, del Pobil AP (2017) Robust motion detection and tracking for human-robot interaction. In: Proceedings of the Companion of the 2017 ACM/IEEE International Conference on Human-Robot Interaction, pp. 401–402. Association for Computing Machinery, New York, NY, USA. 10.1145/3029798.3029799

[2] Gao Y, Huang C-M (2022). Evaluation of socially-aware robot navigation. Front Robotics AI.

[3] Rubagotti M, Tusseyeva I, Baltabayeva S, Summers D, Sandygulova A (2022). Perceived safety in physical human-robot interaction-A survey. Robotics Auton Syst.

[4] Lafuente-Arroyo S, Martin-Martin P, Iglesias-Iglesias C, Maldonado-Bascon S, Acevedo-Rodriguez FJ (2022). RGB camera-based fallen person detection system embedded on a mobile platform. Expert Syst Appl.

[5] Redmon J, Farhadi A (2017) Yolo9000: Better, faster, stronger. In: 2017 IEEE Conference on Computer Vision and Pattern Recognition (CVPR), Honolulu, HI, USA, pp. 6517–6525. 10.1109/CVPR.2017.690

[6] Ramey A, Castro-González A, Malfaz M, Alonso-Martin F, Salichs MA (2017). Vision-based people detection using depth information for social robots: an experimental evaluation. Int J Adv Robotic Syst.

[7] Papadopoulos GT, Axenopoulos A, Daras P (2014) Real-time skeleton-tracking-based human action recognition using kinect data. In: Gurrin C, Hopfgartner F, Hurst W, Johansen H.D, Lee H, O’Connor N.E. (eds.) MultiMedia Modeling. Lecture Notes in Computer Science, vol. 8325, pp. 473–483. Springer, Cham. 10.1007/978-3-319-04114-8_40

[8] Satish P, Jay KB, Amankumar D, Pratik S (2015) Real time skeleton tracking based human recognition system using kinect and arduino, vol. NCRENB 2015. Mumbai, India, pp. 1–6. https://research.ijcaonline.org/ncrenb2015/number2/ncrenb7023.pdf

[9] Paúl R, Aguirre E, García-Silvente M, Muñoz-Salinas R (2012). A new fuzzy based algorithm for solving stereo vagueness in detecting and tracking people. Int J Approx Reason.

[10] Benedek C (2014). 3D people surveillance on range data sequences of a rotating lidar. Pattern Recogn Lett.

[11] Schulz D, Burgard W, Fox D, Cremers AB (2003). People tracking with mobile robots using sample-based joint probabilistic data association filters. Int J Robotics Res.

[12] Arras KO, Mozos OM, Burgard W (2007) Using boosted features for the detection of people in 2D range data. In: Proceedings of the 2007 IEEE International Conference on Robotics and Automation, Roma, Italy, pp. 3402–3407. 10.1109/ROBOT.2007.363998

[13] Spinello L, Siegwart R (2008) Human detection using multimodal and multidimensional features. In: 2008 IEEE International Conference on Robotics and Automation, Pasadena, CA, USA, pp. 3264–3269. 10.1109/ROBOT.2008.4543708

[14] Weinrich C, Wengefeld T, Schroeter C, Gross HM (2014) People detection and distinction of their walking aids in 2D laser range data based on generic distance-invariant features. In: The 23rd IEEE International Symposium on Robot and Human Interactive Communication, Edinburgh, UK, pp. 767–773. 10.1109/ROMAN.2014.6926346

[15] Zivkovic Z, Krose B (2007) Part based people detection using 2D range data and images. In: 2007 IEEE/RSJ International Conference on Intelligent Robots and Systems, San Diego, CA, USA, pp. 214–219. 10.1109/IROS.2007.4399311

[16] Pantofaru C ROS Leg_detector Package. http://wiki.ros.org/leg_detector

[17] Leigh A, Pineau J, Olmedo N, Zhang H (2015) Person tracking and following with 2D laser scanners. In: Proceedings of the 2015 IEEE International Conference on Robotics and Automation (ICRA), Seattle, WA, USA, pp. 726–733. 10.1109/ICRA.2015.7139259

[18] Mohamed SI, Capitanelli A, Mastrogiovanni F, Rovetta S, Zaccaria R (2020). Detection, localisation and tracking of pallets using machine learning techniques and 2D range data. Neural Comput Appl.

[19] Beyer L, Hermans A, Leibe B (2017). Drow: real-time deep learning-based wheelchair detection in 2-D range data. IEEE Robotics Autom Lett.

[20] Beyer L, Hermans A, Linder T, Arras KO, Leibe B (2018). Deep person detection in two-dimensional range data. IEEE Robotics Autom Lett.

[21] Aparicio-Navarro FJ, Kyriakopoulos KG, Parish DJ (2014) Automatic dataset labelling and feature selection for intrusion detection systems. In: 2014 IEEE Military Communications Conference (MILCOM 2014). IEEE Military Communications Conference, pp. 46–51, Baltimore, MD, USA. 10.1109/MILCOM.2014.17

[22] Kim J, On B, Lee I (2021). High-quality train data generation for deep learning-based web page classification models. IEEE Access.

[23] Liang G, Luo Q, Jia Y (2018). Automatic labeling framework for wearable sensor-based human activity recognition. Sensors Mater.

[24] Aguirre E, García-Silvente M, Pascual D (2016) A multisensor based approach using supervised learning and particle filtering for people detection and tracking. In: Reis L.P, Moreira A.P, Lima P.U, Montano L, Muñoz-Martinez V. (eds.) Robot 2015: Second Iberian Robotics Conference. Advances in Intelligent Systems and Computing, vol. 418, pp. 645–657. Springer, Cham. 10.1007/978-3-319-27149-1_50

[25] Aguirre E, García-Silvente M (2019). Using a deep learning model on images to obtain a 2D laser people detector for a mobile robot. Int J Comput Intell Syst.

[26] Huang J, Rathod V, Sun C, Zhu M, Korattikara A, Fathi A, Fischer I, Wojna Z, Song Y, Guadarrama S, Murphy K (2017) Speed/accuracy trade-offs for modern convolutional object detectors. In: 2017 IEEE Conference on Computer Vision and Pattern Recognition (CVPR), pp. 3296–3297. IEEE Computer Society, Honolulu, HI, USA. 10.1109/CVPR.2017.351

[27] Zhou X, Wang D, Krähenbühl P Objects as Points. https://github.com/xingyizhou/CenterNet

[28] Lin T, Maire M, Belongie S, Hays J, Perona P, Ramanan D, Dollár P, Zitnick CL (2014) Microsoft COCO: Common objects in context. In: Fleet D, Pajdla T, Schiele B, Tuytelaars T. (eds.) Computer Vision – ECCV 2014. Lecture Notes in Computer Science, vol. 8693, pp. 740–755. Springer, Cham. 10.1007/978-3-319-10602-1_48

[29] Girshick R, Donahue J, Darrell T, Malik J (2014) Rich feature hierarchies for accurate object detection and semantic segmentation. In: Proceedings of the 2014 IEEE Conference on Computer Vision and Pattern Recognition (CVPR), Columbus, OH, USA, pp. 580–587. 10.1109/CVPR.2014.81

[30] Girshick R (2015) Fast R-CNN. In: 2015 IEEE International Conference on Computer Vision (ICCV), Santiago, Chile, pp. 1440–1448. 10.1109/ICCV.2015.169

[31] Ren S, He K, Girshick R, Sun J (2015) Faster R-CNN: Towards real-time object detection with region proposal networks. In: Proceedings of the 28th International Conference on Neural Information Processing Systems. NIPS’15, vol. 1, pp. 91–99. MIT Press, Cambridge, MA, USA. http://papers.nips.cc/paper/5638-faster-r-cnn-towards-real-time-object-detection-with-region-proposal-networks

[32] He K, Gkioxari G, Dollár P, Girshick R (2017) Mask R-CNN. In: 2017 IEEE International Conference on Computer Vision (ICCV), Venice, Italy, pp. 2980–2988. 10.1109/ICCV.2017.322

[33] Dai J, Li Y, He K, Sun J (2016) R-FCN: Object detection via region-based fully convolutional networks. In: Proceedings of the 30th International Conference on Neural Information Processing Systems. NIPS’16, pp. 379–387. Curran Associates Inc., Red Hook, NY, USA. https://proceedings.neurips.cc/paper/2016/file/577ef1154f3240ad5b9b413aa7346a1e-Paper.pdf

[34] Cai Z, Vasconcelos N (2018) Cascade R-CNN: Delving into high quality object detection. In: 2018 IEEE/CVF Conference on Computer Vision and Pattern Recognition, Salt Lake City, UT, USA, pp. 6154–6162. 10.1109/CVPR.2018.00644

[35] Redmon J, Divvala S, Girshick R, Farhadi A (2016) You only look once: Unified, real-time object detection. In: 2016 IEEE Conference on Computer Vision and Pattern Recognition (CVPR), Las Vegas, NV, USA, pp. 779–788. 10.1109/CVPR.2016.91

[36] Redmon J, Farhadi A YOLOv3: An Incremental Improvement. https://pjreddie.com/darknet/yolo/

[37] Bochkovskiy A, Wang C, Liao H.M YOLOv4: Optimal Speed and Accuracy of Object Detection. https://github.com/AlexeyAB/darknet

[38] Jocher G, Stoken A, Borovec J, NanoCode012, Chaurasia A, TaoXie, Changyu L, V A, Laughing, tkianai, yxNONG, Hogan A, lorenzomammana, AlexWang1900, Hajek J, Diaconu L, Marc, Kwon Y, oleg, wanghaoyang0106, Defretin Y, Lohia A, ml5ah, Milanko B, Fineran B, Khromov D, Yiwei D, Doug, Durgesh, Ingham F ultralytics/yolov5: V5.0 - YOLOv5-P6 1280 Models, AWS, Supervise.ly and YouTube Integrations. 10.5281/zenodo.4679653

[39] Liu W, Anguelov D, Erhan D, Szegedy C, Reed S, Fu C-Y, Berg AC (2016) SSD: Single shot multibox detector. In: European Conference on Computer Vision – ECCV 2016. Lecture Notes in Computer Science, vol. 9905, pp. 21–37. Springer, Cham. 10.1007/978-3-319-46448-0_2

[40] Zhou X, Zhuo J, Krähenbühl P (2019) Bottom-up object detection by grouping extreme and center points. In: 2019 IEEE/CVF Conference on Computer Vision and Pattern Recognition (CVPR), Long Beach, CA, USA, pp. 850–859. 10.1109/CVPR.2019.00094

[41] Cao Z, Hidalgo G, Simon T, Wei S, Sheikh Y (2021). Openpose: realtime multi-person 2D pose estimation using part affinity fields. IEEE Trans Pattern Anal Mach Intell.

[42] Cao Z, Simon T, Wei S, Sheikh Y (2017) Realtime multi-person 2D pose estimation using part affinity fields. In: 2017 IEEE Conference on Computer Vision and Pattern Recognition (CVPR), Honolulu, HI, USA, pp. 1302–1310. 10.1109/CVPR.2017.143

[43] Newell A, Yang K, Deng J (2016) Stacked hourglass networks for human pose estimation. In: Leibe B, Matas J, Sebe N, Welling M. (eds.) Computer Vision – ECCV 2016. Lecture Notes in Computer Science, vol. 9912, pp. 483–499. Springer, Cham. 10.1007/978-3-319-46484-8_29

[44] Chen Y, Song B, Zeng Y, Du X, Guizani M (2021) A deep learning-based approach for fault diagnosis of current-carrying ring in catenary system. Neural Computing and Applications. 10.1007/s00521-021-06280-4

[45] Yuan L, Mao J, Zheng H (2020) Ear detection based on CenterNet. In: 2020 IEEE 2nd International Conference on Civil Aviation Safety and Information Technology (ICCASIT), Weihai, China, pp. 349–353. 10.1109/ICCASIT50869.2020.9368856

[46] Sun Y, Li Z, Wang L, Zuo J, Xu L, Li M (2021) Automatic detection of vehicle targets based on centernet model. In: 2021 IEEE International Conference on Consumer Electronics and Computer Engineering (ICCECE), Guangzhou, China, pp. 375–378. 10.1109/ICCECE51280.2021.9342498

[47] Szegedy C, Ioffe S, Vanhoucke V, Alemi AA (2017) Inception-v4, inception-resnet and the impact of residual connections on learning. In: Proceedings of the Thirty-First AAAI Conference on Artificial Intelligence, vol. 31. San Francisco, California, USA, pp. 4278–4284. 10.1609/aaai.v31i1.11231

[48] Tan M, Pang R, Le QV (2020) Efficientdet: Scalable and efficient object detection. In: 2020 IEEE/CVF Conference on Computer Vision and Pattern Recognition (CVPR), pp. 10778–10787. IEEE Computer Society, Los Alamitos, CA, USA. 10.1109/CVPR42600.2020.01079

[49] Ahmed I, Ahmad M, Rodrigues J, Jeon G (2021). Edge computing-based person detection system for top view surveillance: Using CenterNet with transfer learning. Appl Soft Comput.

[50] Zhang Q, Zhu Z, Bai Y, Liao G, Liu T (2022). Distracted driving detection based on the improved CenterNet with attention mechanism. Multim Tools Appl.

[51] Adept-MobileRobots: Performance PeopleBot Robot. https://www.generationrobots.com/media/PeopleBot-PPLB-RevA.pdf

[52] Intelligence S.S Sick Sensor Intelligence, LMS200. http://www.mysick.com

[53] Bouguet JY Camera Calibration Toolbox for Matlab. http://robots.stanford.edu/cs223b04/JeanYvesCalib/index.html

[54] Chung W, Kim H, Yoo Y, Moon C-B, Park J (2012). The detection and following of human legs through inductive approaches for a mobile robot with a single laser range finder. IEEE Trans Indus Electron.

[55] Kalman RE (1960). A new approach to linear filtering and prediction problems. J Basic Eng.

[56] Kuhn HW (1955). The Hungarian method for the assignment problem. Naval Res Logistics Q.

[57] Frank E, Hall MA, Witten IH (2016) The WEKA Workbench. In: Morgan Kaufmann F.E. (ed.) Data Mining: Practical Machine Learning Tools and Techniques. https://www.cs.waikato.ac.nz/ml/weka/Witten_et_al_2016_appendix.pdf

[58] Leigh A, Pineau J, Olmedo N, Zhang H Leg Tracker. https://github.com/angusleigh/leg_tracker

[59] Bernardin K, Stiefelhagen R (2008). Evaluating multiple object tracking performance: the CLEAR MOT metrics. EURASIP J Image Video Process.

